# Understanding the Development of Chinese EFL Student–Teachers’ Pedagogical Content Knowledge

**DOI:** 10.3389/fpsyg.2021.627728

**Published:** 2021-02-05

**Authors:** Shuo Li, Liyan Liu, Anne Li Jiang

**Affiliations:** School of Foreign Languages, Northeast Normal University, Changchun, China

**Keywords:** pedagogical content knowledge development, developmental trajectories, influencing factors, teacher education programs, EFL student–teachers

## Abstract

Efforts to improve student–teacher education have recently focused on developing adequate Pedagogical Content Knowledge (PCK) as a critical element for effective preparation. Despite many initiatives implemented in teacher education programs, however, their effectiveness in developing student–teachers’ PCK and factors affecting the PCK development are under-researched and evidenced. Drawing upon theories about and research on PCK, this study examined whether a recently updated 2-year teacher education program could develop student–teachers’ PCK effectively and explored what factors influencing the PCK development of student–teachers with different developmental trajectories. Forty English-as-foreign-language (EFL) student–teachers on the program were involved as participants. This study employed a longitudinal research design. Data were collected at four different stages along with the program through the content representation matrix, interviews with all the participants, and focus group interviews with four particularly sampled participants. Findings revealed that the current teacher education program successfully enhanced student–teachers’ PCK and the factors influencing different PCK developmental trajectories were varied and personalized. Implications for teacher education are also discussed.

## Introduction

Pedagogical content knowledge (PCK) in the field of teacher education and professional development has attracted considerable attention as it has long been regarded as an important predictor of teacher quality (Nilsson and Loughran, 2012; Park and Suh, 2015). PCK is central to quality teaching as it reveals the special amalgam of content and pedagogy that forms teachers’ professional understanding of how particular topics, issues or questions are organized, sequenced and represented for teaching (Shulman, 1987). Acquiring appropriate knowledge of content and a rich teaching repertoire that mediates learning takes considerable efforts on the part of individual student–teachers. Teacher education programs can initiate and support this content and repertoire acquisition. However, many teacher education programs are still packed with general information student–teachers “need” to know, provide teachers with “methods” courses relating to teaching a specific subject and teaching practicum in one or more schools (Zhang and Ben Said, 2014; Kind, 2019). Despite repeated calls for change and reform in teacher education, “inadequate subject matter preparation” and “inconsistent teacher education practice” remain long-standing themes (Kind, 2019; Russell and Martin, 2014). Thus, the challenge remains of how to innovate teacher education programs so as to promote student–teachers’ PCK development.

Recently, most programs for teacher education around the world have recognized the importance of cultivating PCK, subject matter knowledge, and general pedagogical knowledge in preparing high-quality teachers (Sickel and Friedrichsen, 2013). For instance, in Finland, facing a shortage of high-quality teachers, teacher education programs are being innovated to equip teachers with strong subject matter knowledge and pedagogical thinking skills, and to prepare teachers to manage the intricate teaching process in a diagnostic manner (Hammerness et al., 2017). There is also a practical turn to increase the amount of practical training and learning in schools (Murray, 2016), which leads many programs to extend the practicum for student–teachers, such as programs in the University of Toronto in Canada, the University of Melbourne in Australia, and the Nanyang University in Singapore (Darling-Hammond, 2017). A recurring theme for innovating these education programs is to enhance connections between theory and practice (Darling-Hammond, 2017). However, the effectiveness of these programs in developing student–teachers’ PCK is still under-researched.

Apart from that, there is limited evidence illustrating the effectiveness of teacher education programs in developing EFL student–teachers’ PCK. Research on tracing student–teachers’ PCK development have been conducted across diverse contexts by a wide range of researchers. Most of them have focused on the domains of science and mathematics (Kind, 2009; Schneider and Plasman, 2011; Depaepe et al., 2013). However, research on EFL student–teachers’ PCK development is scarce (Evens et al., 2016). In fact, such research is imperative, considering the disciplinary characteristics and uniqueness of EFL teaching. First, in contrast to the domains of science and mathematics, where the content is discipline-based throughout, the language in EFL classrooms functions as both the subject matter and the medium that communicates and instructs (Larsen-Freeman and Cameron, 2008; König et al., 2016). Second, domains of science and mathematics are characterized by paradigmatic knowledge, while English is an area that is largely defined by narrative ways of knowledge, which requires EFL teachers to have specific methods and strategies to create simulated communicative environment and opportunities for students to develop content-related skills (Borg, 2006; Gao and Zhang, 2020). Third, distinguished from other domains, in addition to content-specific knowledge, EFL teachers also need to integrate more advanced knowledge of intercultural communication skills into their teaching (Tarone and Allwright, 2005; Yang, 2021). Given these distinct characteristics, any attempt to understand how EFL student–teachers develop PCK and what factors contribute to their PCK development would be undoubtedly necessary.

English-as-foreign-language teaching has a long history in China, but how to prepare well-qualified EFL teachers remains a long-standing challenge for teacher education efforts. In 2014, the MOE of China initiated the landmark *Excellent Teacher Training Program*. This national initiative calls for high-quality professional teacher education programs that promise to cultivate a well-prepared teaching force with solid professional knowledge and rich practical teaching experience. In response, many universities have launched or updated their teacher education programs highlighting the integration of theory and practice. As one of the top-tier normal universities in China, X Normal University’s 2-year postgraduate program is a representative of such initiatives. In X Normal University, EFL student–teachers are the first pilot cohort of this updated program. Whether and how this program develops EFL student–teachers’ PCK can be an important indicator for its effectiveness. As such, this study aims to investigate the effectiveness of this program in developing EFL student–teachers’ PCK and explore factors influencing the PCK development of student–teachers. It aims to answer the following research questions:

(1) Was the current teacher education program effective in enhancing EFL student–teachers’ PCK development?
(2) What factors influenced the PCK development of student–teachers with different developmental trajectories?

## Literature Review

### PCK and Sources for Its Development

Pedagogical content knowledge is a category of teacher knowledge that concerns how teachers transmit their understanding of disciplinary content into forms that are accessible and attainable to their learners (Shulman, 1986, 1987). Since Shulman (1986) first introduced PCK into the field of teacher education, it has been a seductive notion that has drawn many researchers to explore how it might be better elicited and developed. Grossman’s (1990) research identified a variety of sources from which teachers could construct their knowledge of teaching a specific subject. These sources include prior experience as students, subject matter knowledge, professional coursework, and actual classroom practice.

Most of these sources have been supported by previous research. For instance, Halim and Meerah (2002) provided evidence that the possession of adequate subject matter knowledge is a prerequisite for teachers to develop effective PCK as they found that student–teachers with limited subject matter knowledge tend to repeat their misunderstandings and have difficulties in transforming content accurately to the students. This idea was also supported by the work of Smith and Banilower (2015), who posited that incorporating incorrect content into the planning and teaching would inevitably amount to inadequate PCK.

However, Davis (2004) carried out a descriptive study of a primary science student–teacher’s efforts to teach light. She found that though sometimes the student–teacher understood the content well, her instruction flawed. This indicates that adequate subject matter knowledge is not necessarily a guarantee for satisfactory PCK. Actually, while professional courses which emphasize theory learning can enhance student–teachers’ PCK (Kleickmann et al., 2013; Blömeke et al., 2017; Torbeyns et al., 2019), the idea that PCK as a dynamic and flexible entity only becomes meaningful in the classroom context (Park and Oliver, 2008; Alonzo and Kim, 2016; Evens et al., 2018) points to the equal importance of school-based teaching practice in enhancing student–teachers’ PCK.

### The Effectiveness of Teacher Education Programs

In most countries, teacher education programs vary widely in terms of their emphasis on teacher knowledge, program length, academic level, and organization (Zeichner and Conklin, 2005). However, the effectiveness of these programs in promoting student–teachers’ knowledge development is far from conclusive. For instance, Nakiboǧlu et al. (2010) examined the impact of a teacher education program upon nine chemistry student–teachers’ PCK development. Through evaluating student–teachers’ PCK at different stages, they found that “theory” imparted in the course was detached from school “practice.” In another research, Karal and Alev (2016) investigated the development of physics student–teachers’ PCK during their last three academic terms of the teacher education program, which mainly offered methodology courses and teaching training in schools. Their findings showed that the participants manifested declines in subject matter knowledge, improvement in instructional varieties, and increases in learner knowledge. Thus, their research indicates that the teacher education program actually failed to develop student–teachers’ subject matter knowledge effectively.

Another exemplar research examining the teacher education effectiveness was conducted by Mesci et al. (2020), who traced two science student–teachers’ PCK development following a 13-month teacher development program and explored the factors contributing to their PCK development. The findings indicated that the two student–teachers improved their understanding of specific science topics, and successfully enacted their PCK for teaching middle-level science topics. Their research reflected that the effectiveness of the program was influenced by factors such as student–teachers’ self-efficacy, lesson planning, and general pedagogical knowledge.

An important implication drawn from the above studies is that the effectiveness of a teacher education program in developing student–teachers’ PCK is contingent on the program design and multiple factors. It is understood that a program that combines theory learning and school-based practice is more conducive to student–teachers’ PCK development. In our research, the 2-year teacher education program designed by X Normal University is one of such programs. Therefore, this study seeks to examine the effectiveness of this program in developing student–teachers’ PCK and explore the factors influencing their PCK development.

### Approaches to Assessing PCK

To examine the effectiveness of a program in developing student–teachers’ PCK, it is necessary to document their PCK development. However, given the multi-faceted and non-linear nature of PCK (Veal and Makinster, 1999), it is a complex task to capture how it develops over time. Even so, scholars have developed an array of methodologies and techniques for articulating and documenting purposes, including lesson plans (Valk and Broekman, 1999), reflection journals (Gardner and Gess-Newsome, 2011), classroom observations (Rollnick et al., 2008), interviews (Henze et al., 2008), and PCK-tests (Mavhunga and Rollnick, 2013).

A comparatively new instrument of capturing and portraying teachers’ PCK is the content representation (CoRe) matrix developed by Loughran et al. (2004). It includes a series of big ideas about teaching a particular topic and a set of eight pedagogical questions for each row. This instrument firstly requires teachers to select big ideas that are considered essential for students to learn within a particular content area, and then prompts teachers to describe the reasons underlying their pedagogical choices/activities, understandings of their students and ways of assessing students’ learning outcomes (Bertram, 2014). This instrument has been widely applied across diverse contexts by a range of educational researchers to obtain an in-depth understanding of teachers’ PCK (Padilla et al., 2008; Hume and Berry, 2013; Adadan and Oner, 2014; Nilsson and Karlsson, 2018).

In addition to the above qualitative tools, Gess-Newsome et al. (2017) developed a scoring rubric to consider both the quantity and the quality of PCK based on the data from multiple sources, such as interviews, written reflections, lesson plans, among others. Building on Gess-Newsome et al.’s (2017) work, Hanuscin et al. (2018) adapted their rubric and organized it according to the four components of Magnusson et al.’s (1999) PCK model, i.e., knowledge of curriculum, knowledge of learners, knowledge of instructional strategies, and knowledge of assessment. With the new PCK rubric, Hanuscin et al. (2018) assessed teachers’ PCK for a specific topic through analyzing their lesson plans, CoRes, and follow-up interviews, which facilitated comparisons of teachers’ PCK across different groups.

Informed by Hanuscin et al.’s (2018) method, in our study, we first adopted CoRe and interviews to document student–teachers’ PCK, and then scored their responses following the new rubric. This enabled us to compare student–teachers’ PCK across different stages of the program.

## Materials and Methods

### Context

This study focused on X Normal University’s 2-year postgraduate program that was launched in 2017. This program comprises two-semester basic learning, including university-based learning plus practicum in local schools, and an additional 2-month school residency practicum. Student–teachers are encouraged and guided to engage in research and reflection throughout the whole process. During the basic learning stage, student–teachers follow a “3 + 2” learning model, in which they have 3 days learning in the university and 2 days having practicum in local schools under the collaborative supervision of university teachers and school mentors. Guided by the university teachers in lesson preparation, teaching and after-teaching reflection, student–teachers are trained to transfer theoretical knowledge into teaching practices. After that, they will spend 2 months doing residency practicum in schools under the supervision of only school mentors. At this stage, each student–teacher is assigned to one mentor teacher at the practicum school and is supposed to teach at least 10 lessons with their mentor’s instruction. Such an immersive model provides them with plenty of opportunities to observe mentor teachers’ classroom teaching and management and to have hand-on experiences of working as real school teachers. Overall, the program is designed in such a manner as to develop student–teachers’ PCK through a close integration of postgraduate coursework and teaching practicum in partner schools.

### Participants

The participants of this study were a group of EFL student–teachers (*n* = 40) who attended X Normal University’s program from 2017 to 2019. They were all females except one, and their average age was 25.4 years (*SD* = 0.67; range 24–27). These student–teachers attended the program with varied educational backgrounds, about 17.5% of them (*n* = 7) have practical teaching experience before the program, and 12.5% of them (*n* = 5) have engaged in the theoretical training in education. All the EFL student–teachers participated in the first part of data collection. Afterward, four student–teachers were selected through purposeful sampling to participate in the second part of data collection. Their demographic information was shown in Table 1.

**TABLE 1:** The demographic information of the four selected participants.

Participant *pseudonyms	Sex Age	Undergraduate degree	Practical teaching experience	Theoretical training in education
Carol	Female 25	BA in English Teacher education	Micro-teaching practice	Yes
Tara	Female 27	BA in English Teacher Education	Secondary school teaching practice	Yes
Nina	Female 25	BA in Business English	None	No
Zoe	Female 26	BA in English Language and Literature	None	No

### Data Collection

**FIGURE 1:**
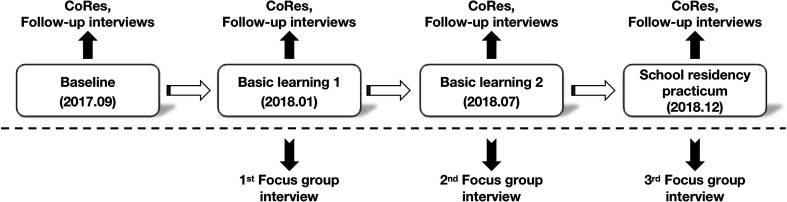
Data collection timeline and data sources.

The data collection procedure consisted of two parts. At first, the data reflecting student–teachers’ PCK development along with the progress of the program were collected. This part lasted for approximately 16 months, during which we collected data for four times: at the baseline (i.e., the beginning of the program), at the end of the first semester’s basic learning, at the end of the second semester’s basic learning, and at the end of the school residency practicum (see the timeline for data collection in Figure 1). Each time we asked all the 40 participants to complete the CoRe matrix based on a chosen topic of teaching. As shown in Table 2, the CoRe is a matrix that includes a series of big ideas about a particular content area and a set of eight pedagogical questions corresponding to the four specific components of PCK. There was a general alignment between each PCK dimension and CoRe framed questions. For instance, Question 1, 2, 3 can be used to elicit teachers’ knowledge of curriculum (KOC), Question 2, 3, 5 for knowledge of learners (KOL), Question 4, 6, 7 for knowledge of instructional strategies (KOIS), and Question 8 for knowledge of assessment (KOA).

**TABLE 2:** Content representation (CoRe) matrix (Loughran et al., 2004).

Content Area: Year level for which this CoRe is Designed	Big idea 1	Big idea 2	Big idea 3	…
(1) What do you intend the students to learn about this idea?				
(2) Why is it important for students to know this?				
(3) What else do you know about this idea that you do not intend students to know yet?				
(4) What difficulties/limitations are connected with teaching this idea?				
(5) What do you know about students thinking which influence teaching about this idea?				
(6) Are there any other factors that influence your teaching of this idea?				
(7) What teaching procedures would you use, and why, for this idea?				
(8) How would you ascertain student understanding of or confusion about this idea?				

Each time after completing the CoRe matrix, we did a follow-up 20-min interview with each participant to further clarify or confirm his or her responses to the CoRe matrix, and elaborate on their knowledge in each PCK domain as elicited by the CoRe. Prior to data collection, the research purpose, the PCK components, and the CoRe matrix were introduced and explained to the participants. During the data collection process, further explanation and assistance in relation to filling in the CoRe matrix were available on demand. In this manner, we hoped to guarantee the accuracy of the matrix data to the best.

The other part of the data was collected through focus group interviews with four purposefully selected participants. After the initial assessment of the 40 student–teachers’ PCK, all participants were ranked according to their PCK scores at the baseline. Four student–teachers, Tara and Carol (pseudonyms) from the first quarter percentile scorers, and Nina and Zoe from the last quarter percentile scorers were selected to participate in three rounds of focus group interviews that occurred respectively at the ends of Semester One, Semester Two and the school residency practicum (see Figure 1). The theme of the interviews was “the factors influencing their PCK development” (see Appendix I for details of the interview protocols). The reason for adopting a focus group interview method is that it allows participants to share and compare their experiences of the program and provided multiple perspectives for the researchers to get rich data (Fontana and Frey, 1994). Each interview lasted about 1 h and was audio-recorded with the participants’ consent. The interview data were transcribed verbatim and translated by the researchers before they were sent back to the participants for verification.

Participation in both parts of data collection was based on the written consent of the participants. Ethics approval of this research was obtained from X Normal University and strictly observed during the whole process of the research.

### Data Analysis

Altogether we received four CoRe responses and four interview transcripts from each participant. We first conducted a holistic reading of each participant’s CoRe responses and the interview transcripts and then coded them according to the alignment between CoRe questions and PCK components respectively. Each CoRe response and interview transcript of a participant would be categorized into four parts corresponding to the four components of PCK. In this way, we built a profile for each participant composed of the identified PCK components at different stages of the program.

We adopted Hanuscin et al.’s (2018) scoring rubric to evaluate all the participants’ responses corresponding to the identified PCK components. This rubric further delineates four sub-components for each PCK domain (see Table 3) and develops a four-level rating scale for each. These four levels are *Limited*, *Basic, Proficient*, and *Advanced* corresponding to the scores from 1 to 4. Using this rubric, each participant’s responses in relation to the PCK components were scored considering the quality of participants’ responses and the quantity of specific information they included. Table 4 shows one example illustrating the scoring process. The purpose of this CoRe question is to evaluate student–teacher’s knowledge of assessment. She used picture prompts and designed increasingly challenging tasks to check students’ understanding. She also identified the advantages of these assessment strategies and explained how these advantages could support students’ learning. According to the rubric, her responses met the criterion of proficient level but failed to meet that of advanced level because she failed to provide opportunities for students’ self-assessment (see Appendix II for the rubric specific for KOA). As such, she received 3 points.

**TABLE 3:** Components and subcomponents of PCK.

(1) Knowledge of curriculum	(2) Knowledge of learners	(3) Knowledge of instructional strategies	(4) Knowledge of assessment
(1.1) Teaching materials	(2.1) Prerequisites: knowledge and skills	(3.1) Subject-specific strategies	(4.1) What to assess
(1.2) Curriculum Standards	(2.2) Common misconceptions	(3.2) Topic-specific strategies: activities	(4.2) Subject-specific assessment strategies
(1.3) Instructional goals	(2.3) Variations in strategies for learning the concepts	(3.3) Topic-specific strategies: representations	(4.3) Topic-specific assessment strategies
(1.4) Sequencing and integrating	(2.4) Sources of student difficulty and common errors	(3.4) Strategies for adapting instruction for diverse learners	(4.4) Purpose of assessment

**TABLE 4:** An extract from student–teacher X’s responses to CoRe and Interview questions.

PCK Domain	CoRe Question	Interview Question
**Knowledge of Assessment**	• **How would you ascertain student understanding of or confusion about this topic? Answer from X student–teacher:** I would use pictures in Disney movies as prompts to check students’ understanding of present continuous tense. Students are asked to fill in the blanks and make complete sentences based on the pictures provided. e.g.: (1) Look, Ariel is______(swim) in the sea, and______(talk) with Sebastian. (2) Rapunzel______(draw) on the wall. (3) What is Snow White doing? …	• **Why do you choose this strategy or activity to assess students’ understanding of this topic? Answer from X student–teacher:** (1) Disney cartoon characters can trigger students’ learning interest. (2) These increasingly challenging tasks can check students’ understanding of the form, meaning and usage of present continuous tense.

In this manner, three independent raters scored all participants’ responses at different stages of the program. The inter-rater reliability was over 70%. Each participant got four sets of PCK component-specific scores, with the possible scores of each set ranging from 4 to 16 points. The total of these four sets of scores (labeled as PCK-total) could range from 16 to 64 points, representing the level of their PCK at the corresponding stage of the program. Informed by Gardner and Gess-Newsome (2011), the PCK-total score (16–64) was divided into four levels labeled as *Limited* (16–27), *Basic* (28–40), *Proficient* (41–52), or *Advanced* (53–64). Then the total of all the 40 participants’ PCK-total scores of each stage was averaged to reflect the general trend of the cohort’s PCK development. To further reveal the effectiveness of this teacher education program in developing student–teachers’ PCK, we used matched pair *t*-tests to compare the participants’ scores of PCK components and PCK-total at different stages.

The focus group interview data were analyzed through a qualitatively inductive process (Strauss and Corbin, 1998). These transcribed interview responses were read and reviewed several times carefully to identify the themes concerning factors influencing student–teachers’ PCK development throughout the program. These themes were then compared, confirmed, and modified within and across these four participants, which led to the final interpretation of the data. During the process of data analysis, the two researchers conducted the coding independently, followed by discussions to reach the inter-rater agreement of over 80%.

## Findings

### The Effectiveness of the Teacher Education Program

**FIGURE 2:**
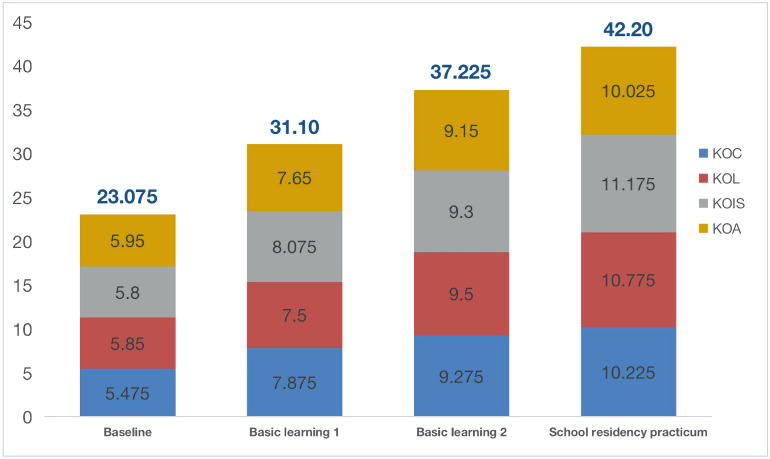
Mean scores for PCK-total and PCK components (*n* = 40). KOC, knowledge of curriculum; KOL, knowledge of learners; KOIS, knowledge of instructional strategies; KOA, knowledge of assessment.

Our first research question concerns the effectiveness of X Normal University’s teacher education program in developing EFL student–teachers’ PCK. As shown in Figure 2, the mean scores of student–teachers’ PCK-total increased from 23.075 to 42.20, indicating that their PCK has been positively influenced by the program. However, student–teachers’ PCK-total mean score at the baseline reported a *Limited* level of PCK, suggesting that their PCK was inadequate and nearly absent at the beginning of the program. One explanation for the initial *Limited* level of PCK may have to do with the lack of practical teaching experience and theoretical training in education before the program. Among the 40 student–teachers, only 17.5% of them (*n* = 7) have practical teaching experience before the program, and 12.5% of them (*n* = 5) have engaged in theoretical training in education. The rest of them may have inadequate procedural knowledge to sequence the presentations of teaching and limited practical experience to handle the intricate process of classroom teaching. Though started with a *Limited* level of PCK, they have made great progress in the following stages of the program, and the growth rates of their PCK-total mean scores for basic learning 1, basic learning 2 and school residency program were 34.8, 19.7, and 13.4% respectively. Especially at the end of the school residency program, student–teachers’ PCK-total mean score has revealed a *Proficient* level of PCK, which implied that they could better organize teaching materials with considerations of students’ learning difficulties, and have more strategies for illustrating certain topics and evaluating students’ outcomes. From baseline to program end, student–teachers’ PCK-total mean scores have indicated a positive shift of their PCK level moving from *Limited* to *Proficient*.

We also conducted three matched pair *t*-tests to examine the changes of PCK-total and PCK components, comparing those scores for PCK-total and PCK components generated at different stages. The results in Table 5 showed that both PCK-total and the four PCK components increased significantly following the program, which confirmed the positive impact of this teacher education program in developing student–teachers’ PCK.

**TABLE 5:** Impact of teacher education program on PCK-total and PCK components.

	KOC		KOL		KOIS		KOA		PCK-total	
Program stages	*M*	*SD*	*M*	*SD*	*M*	*SD*	*M*	*SD*	*M*	*SD*
(1) Baseline	5.475	0.78	5.85	0.73	5.80	0.88	5.95	0.81	23.075	1.71
(2) Basic learning 1	7.875	0.89	7.50	0.84	8.075	1.22	7.65	0.90	31.10	2.22
(3) Basic learning 2	9.275	1.39	9.50	1.21	9.30	1.53	9.15	1.33	37.225	3.49
(4) School residency practicum	10.225	1.45	10.775	1.56	11.175	1.25	10.025	1.44	42.20	3.79
**Matched pair *t*-test 1**	(2) > (1)		(2) > (1)		(2) > (1)		(2) > (1)		(2) > (1)	
*T*	13.460***		10.165***		12.464***		8.119***		24.100***	
effect size	2.86		2.08		2.12		1.98		4.05	
**Matched pair *t*-test 2**	(3) > (2)		(3) > (2)		(3) > (2)		(3) > (2)		(3) > (2)	
*T*	6.445***		9.874***		4.593***		7.293***		14.566***	
effect size	1.15		1.92		0.88		1.32		2.08	
**Matched pair *t*-test 3**	(4) > (3)		(4) > (3)		(4) > (3)		(4) > (3)		(4) > (3)	
*T*	3.485**		4.085***		6.925***		3.176*		7.773***	
effect size	0.67		0.91		1.33		0.63		1.37	

***p < 0.0001; **p < 0.001; *p < 0.01.

Closer inspection of Table 5 revealed that student–teachers’ PCK-total and PCK components increased comparatively faster during the two semesters’ basic learning than in the school residency practicum, especially regarding KOC (Basic learning 2: *M* = 9.275, *SD* = 1.39; School residency practicum: *M* = 10.225, *SD* = 1.45; *t* = 3.485**, effect size = 0.67) and KOA (Basic learning 2: *M* = 9.15, *SD* = 1.33; School residency practicum: *M* = 10.025, *SD* = 1.44; *t* = 3.176*, effect size = 0.63). Only KOIS was an exception to this pattern, as it experienced more growth in school residency practicum than in the second semester’s basic learning (Basic learning 2: *M* = 9.30, *SD* = 1.53; School residency practicum: *M* = 11.175, *SD* = 1.25; *t* = 6.925***, effect size = 1.33). Though KOIS saw less progress in the second semester’s basic learning, student–teachers achieved the best growth in developing KOIS throughout the program (Baseline: *M* = 5.80, *SD* = 0.88; School residency practicum: *M* = 11.175, *SD* = 1.25). However, KOA appeared to be more challenging to most of student–teachers, as it registered the least growth comparing to other three PCK components (Baseline: *M* = 5.95, *SD* = 0.81; School residency practicum: *M* = 10.025, *SD* = 1.44). Summarizing these findings above, we acknowledged that this 2-year teacher education program successfully enhanced student–teachers’ PCK though there was still room for improvement.

### Factors Influencing Student–Teachers’ PCK Development

**FIGURE 3:**
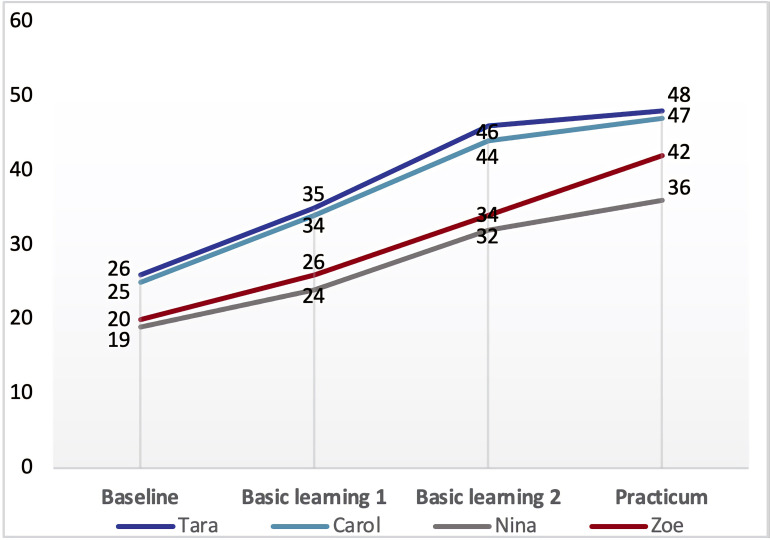
Four student–teachers’ different trajectories of PCK development. KOC, knowledge of curriculum; KOL, knowledge of learners; KOIS, knowledge of instructional strategies; KOA, knowledge of assessment.

The second focus of this research concerns the factors influencing the PCK development of student–teachers with different developmental trajectories. As shown in Figure 3, the four purposefully selected participants’ PCK development demonstrated different trajectories. According to the general developmental trend, we classified their trajectories into three types: surge-stabilized (Tara and Carol), linear-increased (Zoe), and zigzag-progressed (Nina). The analysis of the focus group interview responses revealed common as well as distinctive factors bearing on their PCK development.

### Common Factors: *Well-Designed Courses and Professional Learning Community*

All the four participants reflected that the well-designed courses during the two semesters’ basic learning played an important role in developing their overall PCK, especially regarding the development of curricular knowledge. Courses such as *Analysis of Curriculum Standards*, *Teaching English from Linguistic, Literary, and Intercultural Perspectives*, and *Micro-teaching*, were unanimously considered as valuable, as they not only equipped student–teachers with an in-depth theoretical understanding of the target curriculum standards and a holistic picture of different facets of teaching practice but also provided regulated chances to teach in simulated scenarios, as evident in Nina’s quote:

The Analysis of Curriculum Standards was extremely helpful. The teacher elaborated on the latest requirements for English teaching and the reasons why you teach in certain ways instead of others. It provokes my thoughts on what I’m going to teach and why I teach them in such ways, which I never thought about carefully before the program.

Obviously, the program courses engaged Nina in purposeful consideration of the curriculum standards and the rationale behind teaching practice. Tara expressed that the courses enriched her perspectives on English teaching and consolidated her prior subject matter knowledge, as she said,

I had never thought about why we pronounced words’ plural forms in different ways, but after I took the course Teaching English from a Linguistic Perspective, I could explain it with the “assimilation phenomenon” to my students.

Apart from the courses’ benefits for the improvement of student–teachers’ knowledge of curriculum and knowledge of the subject matter, their overall PCK was also enhanced through simulated teaching, as shown in Zoe’s narrative:

Micro-teaching gave me an “all-inclusive experience,” in which I learned to set up focused teaching objectives, anticipate learning difficulties, adopt proper strategies to present topics and assess students’ learning. These experiences were important for me and prepared me well for the challenges in authentic teaching.

Zoe’s comments on *Micro-teaching* revealed a way of improving student–teachers’ overall PCK through simulated teaching, which helped them prepare well before they embark on real teaching.

Another affecting factor commonly revealed was the professional learning community composed of university supervisors, school mentors and student–teachers. Tara and Carol expressed that participation in the professional learning community during basic learning promoted changes in their PCK, especially concerning knowledge of instructional strategies. They highlighted the facilitating role of the learning community as it prepared them with a full understanding of the content they are learning or teaching, and supplied them with specific strategies for enacting effective teaching, as Tara explicitly expressed,

I enjoyed collaborating with school mentors, university supervisors, and knowledgeable peers, as I could share my teaching design with them, and listen to their ideas on the same topic. This collaborative discussion minimized my confusion on that topic and improved my teaching as the more I understand the content, the better I am in explaining it.

Tara’s experience showed that the collaborative inquiry of teaching helped her gain a better understanding of the teaching content, and enabled her to better present it to her students. Carol added that this collaborative inquiry of teaching also increased her knowledge of specific instructional strategies, as she said,

I developed many sparkling ideas and effective strategies such as analogy, images, metaphors while I was engaged in this collaborative workshop.

Similar descriptions were embodied in Zoe’s responses, as evident in her quote,

I benefited greatly from cooperating teachers’ and peers’ comments on my instruction. They analyzed the strengths and weaknesses of my teaching and told me how to get the content out more effectively with proper activities and effective strategies.

The learning community enhanced Zoe’s knowledge of instructional strategies with pedagogical support and valuable comments from cooperating teachers and peers. In addition, Nina mentioned that observation of mentor teachers’ teaching promoted her understanding of learners’ difficulties, and increased her awareness to assess students’ outcomes, as she expressed,

I rarely looked at whether my students have learned. But I found that my mentor’s teaching was student-centered, and she adopted various strategies to promote students’ participation and check if the students really understood what they were taught.

### Distinctive Factors Affecting the Surge-Stabilized: *Prior Learning Experiences and Mentoring Support*

**FIGURE 4:**
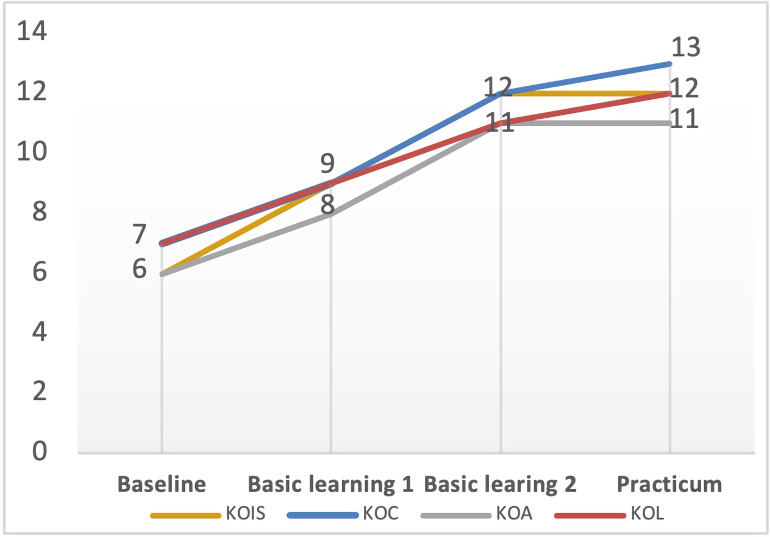
Tara’s PCK developmental trajectory. KOC, knowledge of curriculum; KOL, knowledge of learners; KOIS, knowledge of instructional strategies; KOA, knowledge of assessment.

**FIGURE 5:**
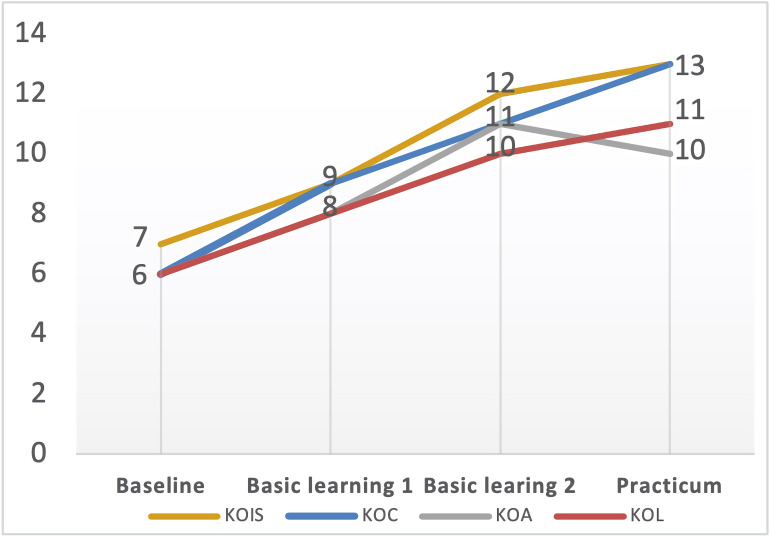
Carol’s PCK developmental trajectory. KOC, knowledge of curriculum; KOL, knowledge of learners; KOIS, knowledge of instructional strategies; KOA, knowledge of assessment.

Tara’s and Carol’s trajectories of PCK development indicated that all their PCK components developed comparatively faster from baseline to the second semester of basic learning and then leveled off in the school residency practicum (see Figures 4, 5). Tara and Carol explained that their prior learning experience in undergraduate teacher education has laid the foundation for their learning and teaching in the program, and thus accelerated the internalization of their curricular knowledge, facilitated their acquisition of new instructional strategies, and equipped them with a basic understanding of learners, as reflected in Carol’s words,

It was something I have learned before that made me feel easier to understand the courses in the program. And in the course Models of Teaching, I learned many useful teaching models such as attaining concepts, role-playing, and memorization.

Carol’s prior learning experiences enabled her to thoroughly understand the university courses and to better learn new models and representations to facilitate students’ learning. Similar to Carol, Tara clearly indicated the importance of these prior teacher education experiences as being important in her development of learner knowledge. She recounted her experiences as below:

I had practical teaching experiences at secondary schools, and these experiences gave me a general understanding of students’ learning difficulties, interest, and developmental level. That understanding probably worked in my favor because I could better recognize students’ needs and sequence proper activities to promote their participation and learning.

However, their PCK did not witness much progress while they were in the school residency practicum, specifically Tara’s knowledge of instructional strategies and Carol’s knowledge of assessment have not developed and even dropped back to the previous level. Tara explained that the main reason for her limited progress on knowledge of instructional strategies was school mentors’ weak mentoring support.

The school mentor assigned to me was really busy dealing with school affairs and did not pay much attention to the use of teaching methods or strategies. Her poor planning of teaching activities, lack of instructional strategies, and ineffective classroom management may not be a good example for me.

Tara’s experience pointed out that mentors may not successfully model all desirable qualities for student–teachers. Her perception of mentors’ modeling function was echoed and supplemented in Carol’s comments:

Some expert teachers I observed greatly relied on the commercially available curriculum materials and lacked assessment strategies targeting students’ learning about topics. The absence of good modeling failed to inform my following instruction concerning assessing students’ learning.

As reflected in Tara’s and Carol’s experiences, it can be inferred that the qualities of mentor teachers also greatly influence their PCK development.

### Distinctive Factors Affecting the Zigzag-Progressed: *Emotion and Subject Matter Knowledge*

**FIGURE 6:**
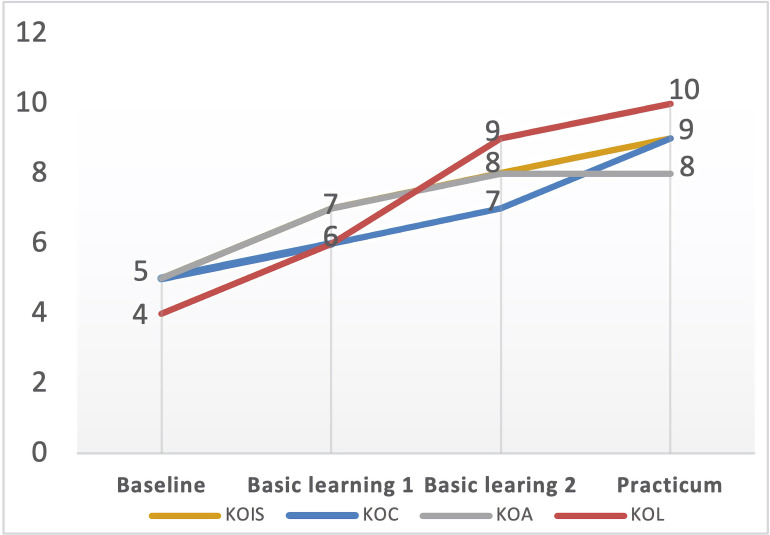
Nina’s PCK developmental trajectory. KOC, knowledge of curriculum; KOL, knowledge of learners; KOIS, knowledge of instructional strategies; KOA, knowledge of assessment.

Nina gained the least growth of PCK compared with the other three cases in the program (see Figure 6). In the interview, she expressed some negative emotions because she felt the university coursework too hard to follow for lack of subject matter knowledge, as indicated in the following quote,

I was overwhelmed by the sheer immensity of the learning tasks, and they were hard for me. For example, the courses such as Teaching English from Linguistic, Literary and Intercultural Perspectives were difficult for me as I knew little about them, though it was interesting to design teaching from these perspectives.

Such negative feelings also wore down her motivation of becoming a teacher, as she expressed,

I feel anxious the night before I’m gonna teach in the real classroom and I can’t even sleep. Maybe I’m not fit for a teacher.

Furthermore, lack of subject matter knowledge affected Nina’s growth in her knowledge of instructional strategies. In the following account, she described how challenging it was to enact teaching with limited subject matter knowledge in English:

*I have rehearsed several times about how to teach that topic in my mind*…, *and I went into the classroom thinking that I could do it well. But it upset me when they didn’t get what I was teaching*…, *as it was a really able class and most of the students were good at English, I knew it was my teaching didn’t get the content out to them. It was embarrassing as I was an English teacher but didn’t know how to explain it in English*…

Apart from knowledge of instructional strategies, her fragmented subject matter knowledge also obstructed the development of her assessment knowledge, as implied in her quotes:

Using the assessment activities and the questions [in the textbooks]has helped me with assessing whether my students learned. However, I’m afraid of making mistakes and rarely use higher-level questions or activities to interact with students.

Nina demonstrated that her knowledge of assessment largely derived from the assessment activities and questions in the textbooks. Although Nina tacitly mentioned some other forms of formative assessment, she refrained from wandering too far away from the textbooks. Again, this is because she was afraid of making mistakes or “embarrassing herself” due to insufficient subject matter knowledge.

### Distinctive Factor Affecting the Linear-Increased: *Motivation*

**FIGURE 7:**
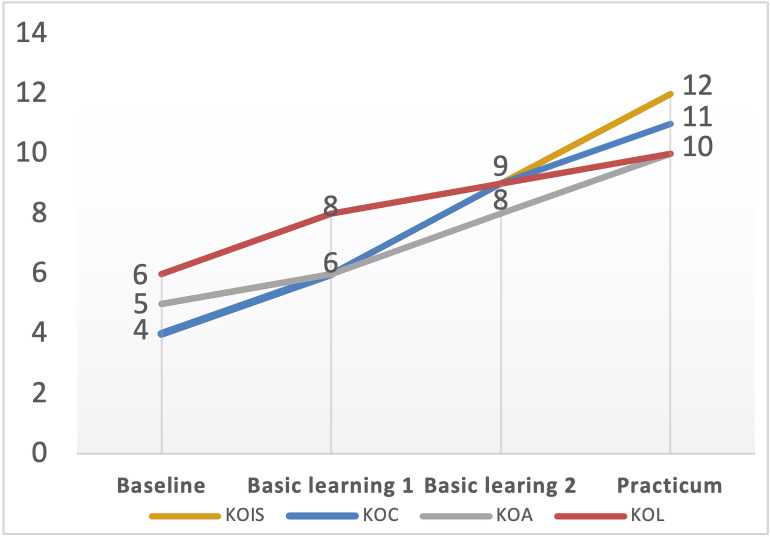
Zoe’s PCK developmental trajectory. KOC, knowledge of curriculum; KOL, knowledge of learners; KOIS, knowledge of instructional strategies; KOA, knowledge of assessment.

Though started with a low level of PCK, Zoe’s story was quite different from Nina’s. There was a consecutive growth in Zoe’s development of PCK (see Figure 7). In addition to the university course learning she mentioned above, she associated her PCK development with her “vision” of being a qualified English teacher. This vision was set up when she won the “Excellent Teaching Design Award” in the 2nd Professional Teaching Skills Competition for M.Ed (Masters of Education), which was held in May 2018. This event can be thought of as a critical incident that drastically affected her life for almost 6 months. Dörnyei et al. (2016) posits this phenomenon of developing self-in-future images, or “vision,” may trigger and propel a directed motivational current (DMC), which is important in forming an individual’s motivational surges for doing the purposeful activities. Caught up in a DMC, Zoe developed some regular routines to achieve her goal. She said,

*I devoted the mornings to reading books on English teaching, took notes while observing expert teachers’ classes, and learned from mentors’ suggestions*…, *and I felt more competent to teach English.*

She added that the experiences during her school residency practicum gave her sustaining motivation in achieving the goal.

I saw several teachers of my age in school. They treated me as a real teacher, and told me how to manage classrooms and build good relationships with students. I progressed a lot with their help, and they told me that if I continue that way, I would be a good teacher.

The above experience helped her feel more prepared and more willing to be an English teacher. Her motivation continued until she was accepted as an English teacher in an excellent school by the end of this program. Zoe’s motivational experience revealed that directed motivational currents propel an individual toward their goal accompanied by positive feelings and enjoyment (Dörnyei et al., 2016; Zarrinabadi and Tavakoli, 2017), and it is important to create conditions for such motivations to be generated and strengthened.

## Discussion and Conclusion

In this study, we investigated the effectiveness of a carefully designed 2-year teacher education program on developing EFL student–teachers’ PCK and explored the factors influencing the PCK development of student–teachers with different developmental trajectories. Specifically, this study presented the following findings: (1) this 2-year teacher education program successfully enhanced EFL student–teachers’ PCK and promoted a positive shift of their PCK level moving from *Limited* to *Proficient*; (2) among the four purposefully sampled participants, we identified three types of PCK developmental trajectories: surge-stabilized (Tara and Carol), linear-increased (Zoe), and zigzag-progressed (Nina). Factors affecting their PCK development included well-designed courses, professional learning communities, prior learning experiences, mentoring support, emotion, subject matter knowledge and motivation. On the whole, given the general recognition that it is challenging to examine the effectiveness of a program since the changes in student–teachers’ knowledge and practices may unfold slowly over time rather than be observable at a point in time (Sword, 2011; Chalmers and Gardiner, 2015), our study managed to reveal student–teachers’ PCK changes across different stages based on the analysis of longitudinal data and testify the effectiveness of a recently updated teacher education program. The findings of this study also provide insights into the research on exploring student–teachers’ PCK, especially in EFL teaching context, as it uncovers distinctive trajectories through which EFL student–teachers develop their PCK and the factors contributing to their PCK development, thus enriching the research on EFL teacher education and professional development.

Specifically, this study shed light on several key aspects. First, the design of a teacher education program may affect the rate of student–teachers’ PCK development. As shown in the quantitative findings, student–teachers’ PCK developed comparatively faster during the two semesters’ basic learning than during the school residency practicum. An important reason is that student–teachers gained more support and guidance from the well-designed courses and the professional learning communities embedded in the two semesters’ basic learning, as is evident in the interview findings. In contrast, though the school residency practicum offered them more opportunities to teach and experience school culture as real teachers, they mostly had to deal with teaching issues on their own. With less professional guidance, their PCK at this stage developed at a relatively slow pace. However, student–teachers’ experiences in school residency practicum mitigated the gap between their ideal teaching environment and reality, and reduced the reality shock that they would experience in the first few years of teaching (Chiang, 2008; Fazio and Volante, 2011).

Second, despite significant growth in PCK as a whole, a much closer look indicates student–teachers’ growth in different PCK components is not even. As shown in our study, KOIS achieved the best growth following the program, while KOA realized the least growth. One possible explanation is that KOIS as a core PCK component has been greatly emphasized both in theory and practice, which could be easily learned and developed through course learning and classroom observation. In contrast to KOIS, KOA was less introduced and implemented in course learning and classroom teaching, which was less available for student–teachers to observe and learn. Moreover, related to this, the focus group interview with four particular participants (especially Nina and Carol) revealed that student–teachers may lack the awareness to assess whether their students have learned, and without good modeling of ways to assess students’ understanding, they made little progress in developing their assessment knowledge.

Third, while acknowledging the overall effectiveness of the program, student–teacher’ trajectories of PCK development vary due to personalized factors. Coinciding with Friedrichsen et al. (2009), our research also recognizes the non-neglectable role of students’ prior learning experiences on their current teaching and learning, as evident in Tara’s and Carol’s cases. Their experiences confirm that memories of past education is an important source of PCK (Haston and Leon-Guerrero, 2008). Their trajectories also indicate that mentoring support may be an important factor influencing the development of PCK as experienced mentors would provide pedagogical supports, targeted feedbacks, model practices and serve as agents of socialization for student–teachers (Riedler and Eryaman, 2016; Alemdağ and Özdemir-Şimşek, 2017). In addition, Nina’s zigzag-progressed trajectory suggests the importance of subject matter knowledge in developing PCK, especially in EFL teaching context. Her story provides evidence that student–teachers with fragmented knowledge in subject matter would easily fall into “embarrassing” situations when they are unable to explain content accurately and effectively (Halim and Meerah, 2002; Smith and Banilower, 2015; Gess-Newsome et al., 2017). Such “embarrassing” situations obviously do not incentivize her motivation of being a teacher and even produce negative emotions obstructing her learning to teach. Comparatively, Zoe demonstrates stronger motivation of learning to teach and set up a vision of being a qualified English teacher, which drives her development of PCK following the program. Her linear-increased trajectory provides evidence to support the finding that student–teachers with higher motivation may be more willing to engage in various activities that stretch their teaching abilities than those with lower motivation (Thomson et al., 2017).

Apart from these personalized factors, the analysis of the focus group interview responses also reveals common factors bearing on student–teachers’ PCK development. For one thing, student–teachers’ PCK development is primarily attributable to well-designed courses, which is in line with Evens et al.’s (2017) research on PCK which showed that more courses on PCK lead to more PCK development. For another, our findings also support Dogan et al.’s (2016) claim that professional learning communities could promote changes in teachers’ knowledge and practices.

A primary limitation of this study lies in our focus on student–teachers’ reported PCK. Since self-report may not always be consistent with real classroom teaching practice, future research should explore whether student–teachers’ reported PCK agree with their enacted PCK in EFL teaching context. Regarding the implications for teacher education, our research makes the following suggestions. First, simply advocating more time for teaching practice as a means of preparing student–teachers is not the answer to creating better teachers (Donaldson, 2011). Instead, professional guidance and support should permeate all the practical experiences to ensure the quality of student–teachers’ learning and teaching. Second, language teachers need to be equipped with both general and language specific knowledge of teaching (Li, 2020). Thus, more courses illustrating the specific features of the target language (e.g., linguistics, literature, and intercultural communications) are necessary to promote EFL student–teachers’ acquisition of PCK. Third, mentor support is vital in teacher education because of mentor teachers’ central role in modeling and disseminating ideas of teaching and learning (Peercy and Sharkey, 2020). Hence, school mentor teachers should be carefully selected from those who are experts in their subject area and are willing to provide student–teachers with valuable feedback, careful coaching, and professional career advice. Finally, motivation influences what a teacher learns and chooses to implement in practice (Yuan and Zhang, 2017). It is imperative for teacher education programs to create a motivating environment to promote learning engagement and significant growth in student–teachers’ PCK. These implications make sense for similar programs in other parts of the world, and highlight that more research is needed on how education programs can better promote EFL student–teachers’ PCK development.

## Data Availability Statement

The raw data supporting the conclusions of this article will be made available by the authors, without undue reservation.

## Ethics Statement

The studies involving human participants were reviewed and approved by Graduate School of Northeast Normal University. The patients/participants provided their written informed consent to participate in this study. Written informed consent was obtained from the individual(s) for the publication of any potentially identifiable images or data included in this article.

## Author Contributions

All authors listed have made a substantial, direct and intellectual contribution to the work, and approved it for publication.

## Conflict of Interest

The authors declare that the research was conducted in the absence of any commercial or financial relationships that could be construed as a potential conflict of interest.

## Appendix I

Focus group interview protocols.

(1) Do you have any new understanding concerning the curricular knowledge? What are the sources of your understanding?

(2) Do you know your students well? What contributes to your understanding of your students?

(3) Have you developed any new strategies to facilitate students’ learning? How do you acquire them?

(4) Have you assessed your students’ learning? How do you know these ways of assessment?

(5) What promotes or obstructs your developing of PCK? Please give some examples.

Overall, are you satisfied with the program? What should be improved to achieve better learning and teaching?

## Appendix II

PCK scoring rubric sample.

**Table:**

Teacher		Topic	Reviewer	PCK score
Indicators	Limited-1	Basic-2	Proficient-3	Advanced-4
What to assess	What is being assessed is not consistent with what is being taught, the standards identified. etc.	Teacher ideas about dimensions of student learning to assess are tacit. Assessment overall is aligned with the lesson goals but may overlook some key dimension(s).	Teacher identifies appropriate dimensions to assess but has limited rationale for why that is appropriate. Assessment overall is aligned with goals of the lesson.	Teacher can articulate what to assess and explain why that dimension is appropriate to assess. Assessment is comprehensive to the lesson’s learning goals.
Subject-specific strategies	Teacher relies on general assessment strategies rather than subject-specific assessment strategies for subject.	Teacher utilizes subject-specific strategies but does not consider advantages and disadvantages of particular strategies.	Teacher selects from among subject specific strategies for assessing English learning by considering advantages and disadvantages of each.	Teacher implements a variety of subject-specific strategies for assessing English learning while considering advantages and disadvantages of each. Strategies provide opportunities for student self-assessment.
Topic-specific Strategies	Teacher relies on general assessment strategies rather than topic-specific assessment strategies.	Teacher utilizes topic-specific strategies but does not consider advantages and disadvantages of particular strategies.	Teacher selects from among topic specific strategies for assessing English learning by considering advantages and disadvantages of each.	Teacher implements a variety of topic-specific strategies for assessing English learning while considering advantages and disadvantages of each. Strategies provide opportunities for student self-assessment.
Purpose of assessment (when and why to assess)	Teacher implements assessment for limited purposes (e.g., summative assessment only) and/or misses opportunities to assess throughout the lesson.	Teacher implements assessment at different points in the lesson but may not capitalize on assessment for multiple purposes (e.g., formative assessment is not used to inform instructions).	Teacher implements assessment throughout the lesson for multiple purposes (e.g., formative and summative).	Teacher implements assessment at appropriate time throughout the lesson to meet multiple purposes (e.g., summative, formative, evaluative, and educative.)

*Evidence: CoRes, and Follow-up interviews.*

## References

[B1] Adadan E. Oner D. (2014). Exploring the progression in pre-service che-mistry teachers’ pedagogical content knowledge representations: the case of “behaviour of gases”. *Res. Sci. Educ.* 44 829–858. 10.1007/s11165-014-9401-6

[B2] Alemdağ E. Özdemir-Şimşek P. (2017). Pre-service teachers’ evaluation of their mentor teachers, school experiences, and theory-practice relationship. *Int. J. Prog. Educ.* 13 165–179.

[B3] Alonzo A. C. Kim J. (2016). Declarative and dynamic pedagogical content knowledge as elicited through two video-based interview methods. *J. Res. Sci. Teach.* 53 1259–1286. 10.1002/tea.21271

[B4] Bertram A. (2014). CoRes and PaP-eRs as a strategy for helping beginning primary teachers develop their pedagogical content knowledge. *Educ. Química* 25 292–303. 10.1016/S0187-893X(14)70545-2

[B5] Blömeke S. König J. Busse A. Döhrmann M. Hoth J. (2017). Professional competencies of (prospective) mathematics teachers-cognitive versus situated approaches. *Educ. Stud. Math.* 94 161–182. 10.1007/s10649-016-9713-8

[B6] Borg S. (2006). The distinctive characteristics of foreign language teachers. *Lang. Teach. Res.* 10 3–31. 10.1191/1362168806l82oa

[B7] Chalmers D. Gardiner D. (2015). An evaluation framework for identifying the effectiveness and impact of academic teacher development programmes. *Stud. Educ. Eval.* 46 81–91. 10.1016/j.stueduc.2015.02.002

[B8] Chiang M. H. (2008). Effects of fieldwork experience on empowering prospective foreign language teachers. *Teach. Teach. Educ.* 24 1270–1287. 10.1016/j.tate.2007.05.004

[B9] Darling-Hammond L. (2017). Teacher education around the world: what can we learn from international practice? *Eur. J. Teach. Educ.* 40 291–309. 10.1080/02619768.2017.1315399

[B10] Davis E. A. (2004). Knowledge integration in science teaching: analysis of teachers’ knowledge development. *Res. Sci. Educ.* 34 21–53. 10.1023/B:RISE.00000

[B11] Depaepe F. Verschaffel L. Kelchtermans G. (2013). Pedagogical content knowledge: a systematic review of the way in which the concept has pervaded mathematics educational research. *Teach. Teach. Educ.* 34 12–25. 10.1016/j.tate.2013.03.001

[B12] Dogan S. Pringle R. Mesa J. (2016). The impacts of professional learning communities on science teachers’ knowledge, practice and student learning: a review. *Prof. Dev. Educ.* 42 569–588. 10.1080/19415257.2015.1065899

[B13] Donaldson G. (2011). *Teaching Scotland’s Future: A Report of the Review of Teacher Education in Scotland.* Edinburgh: Scottish Government.

[B14] Dörnyei Z. Henry A. Muir C. (2016). *Motivational Currents in Language Learning: Frameworks for Focused Interventions.* New York, NY: Routledge.

[B15] Evens M. Elen J. Depaepe F. (2016). Pedagogical content knowledge in the context of foreign and second language teaching: a review of the research literature. *Porta Linguarum* 26 187–200.

[B16] Evens M. Elen J. Depaepe F. (2017). Effects of opportunities to learn in teacher education on the development of teachers’ professional knowledge of French as a foreign language. *Journal of Advances in Education Research* 2 265–279.

[B17] Evens M. Tielemans K. Elen J. Depaepe F. (2018). Pedagogical content knowledge of French as a foreign language: differences between pre-service and in-service teachers. *Educ. Stud.* 45 422–439. 10.1080/03055698.2018.1446339

[B18] Fazio X. Volante L. (2011). Pre-service science teachers’ perceptions of their practicum classrooms. *Teach. Educ.* 46 126–144. 10.1080/08878730.2011.553028

[B19] Fontana A. Frey J. H. (1994). “Interviewing: the art of science,” in *Handbook of Qualitative Research*, eds Denzin N. K. Lincoln Y. S. (Thousand Oaks, CA: Sage), 361–376.

[B20] Friedrichsen P. J. Abell S. K. Pareja E. M. Brown P. L. Lankford D. M. Volkmann M. J. (2009). Does teaching experience matter? Examining biology teachers’ prior knowledge for teaching in an alternative certification program. *J. Res. Sci. Teach.* 46 357–383. 10.1002/tea.20283

[B21] Gao L. X. Zhang L. J. (2020). Teacher learning in difficult times: examining foreign language teachers’ cognitions about online teaching to tide over COVID-19. *Front. Psychol.* 11:549653 10.3389/fpsyg.2020.549653 PMC7533586 33071866

[B22] Gardner A. L. Gess-Newsome J. (2011). “A PCK rubric to measure teachers’ knowledge of inquiry-based instruction using three data sources,” in *Paper Presented at the Annual Meeting of the National Association for Research in Science Teaching*, Orlando, FL.

[B23] Gess-Newsome J. Taylor J. A. Carlson J. Gardner A. L. Wilson C. D. Stuhlsatz M. A. M. (2017). Teacher pedagogical content knowledge, practice, and student achievement. *Int. J. Sci. Educ.* 41 16–36. 10.1080/09500693.2016.1265158

[B24] Grossman P. L. (1990). *The Making of a Teacher: Teacher Knowledge and Teacher Education.* New York, NY: Teacher College.

[B25] Halim L. Meerah S. M. (2002). Science trainee teachers’ pedagogical content knowledge and its influence on physics teaching. *Res. Sci. Technol. Educ.* 2 215–225. 10.1080/0263514022000030462

[B26] Hammerness K. Ahtianinen R. Sahlberg P. (2017). *Empowered Educators in Finland: How High-Performing Systems Shape Teaching Quality.* San Francisco, CA: Jossey-Bass.

[B27] Hanuscin D. Cisterna D. Lipsitz K. (2018). Elementary teachers’ pedagogical content knowledge for teaching the structure and properties of matter. *J. Sci. Teach. Educ.* 29 665–692. 10.1080/1046560X.2018.1488486

[B28] Haston W. Leon-Guerrero A. (2008). Sources of pedagogical content knowledge: reports by preservice instrumental music teachers. *J. Music Teach. Educ.* 17 48–59. 10.1177/1057083708317644

[B29] Henze I. van Driel J. H. Verloop N. (2008). Development of experienced science teachers’ pedagogical content knowledge of models of the solar system and the universe. *Int. J. Sci. Educ.* 30 1321–1342. 10.1080/09500690802187017

[B30] Hume A. Berry A. (2013). Enhancing the practicum experience for pre- service chemistry teachers through collaborative CoRe design with mentor teachers. *Res. Sci. Educ.* 43 2107–2136. 10.1007/s11165-012-9346-6

[B31] Karal I. S. Alev N. (2016). Development of pre-service physics teachers’ pedagogical content knowledge (PCK) throughout their initial training. *Teach. Dev.* 20 162–180. 10.1080/13664530.2015.1124138

[B32] Kind V. (2009). Pedagogical content knowledge in science education: perspectives and potential for progress. *Stud. Sci. Educ.* 45 169–204. 10.1080/03057260903142285

[B33] Kind V. (2019). Development of evidence-based, student-learning-oriented rubrics for pre-service science teachers’ pedagogical content knowledge. *Int. J. Sci. Educ.* 41 911–943. 10.1080/09500693.2017.1311049

[B34] Kleickmann T. Richter D. Kunter M. Elsner J. Besser M. Krauss S. (2013). Teachers’ content knowledge and pedagogical content knowledge: the role of structural differences in teacher education. *J. Teach. Educ.* 64 90–106. 10.1177/0022487112460398

[B35] König J. Lammerding S. Nold G. Rohde A. Strauß S. Tachtsoglou S. (2016). Teachers’ professional knowledge for teaching English as a foreign language: assessing the outcomes of teacher education. *J. Teach. Educ.* 67 320–337. 10.1177/0022497116644956

[B36] Larsen-Freeman D. Cameron L. (2008). *Complex Systems and Applied Linguistics.* Oxford: Oxford University Press.

[B37] Li M. (2020). Multimodal pedagogy in TESOL teacher education: students’ perspectives. *System* 94:102337 10.1016/j.system.2020.102337

[B38] Loughran J. Mulhall P. Berry A. (2004). In search of pedagogical content knowledge in science: developing ways of articulating and documenting professional practice. *J. Res. Sci. Teach.* 41 370–391. 10.1002/tea.20007

[B39] Magnusson S. Krajacik J. Borko H. (1999). “Nature, sources, and development of pedagogical content knowledge for science teaching,” in *Examining Pedagogical Content Knowledge: The Construct and Its Implication for Science Education*, eds Gess-Newsome J. Lederman N. G. (Dordrecht: Kluwer Academic), 95–132. 10.1007/0-306-47217-1_4

[B40] Mavhunga E. Rollnick M. (2013). Improving PCK of chemical equilibrium pre-service teachers. *Afr. J. Res. Math. Sci. Technol. Educ.* 17 113–125. 10.1080/10288457.2013.828406

[B41] Mesci G. Schwartz R. S. Pleasants B. A. (2020). Enabling factors of pre-service science teachers’ pedagogical content knowledge for nature of Science and nature of scientific inquiry. *Sci. Educ.* 29 263–297. 10.1007/s11191-019-00090-w

[B42] Murray J. (2016). “Trends in teacher education across Europe,” in *New Aspects in European Teacher Education*, eds Falus J. Orgoványi-Gaidos J. (Eger: Líceum Kiadó), 6–17.

[B43] Nakiboǧlu C. Karakoç Ö De Jong O. (2010). Examining pre-service chemistry teachers’ pedagogical content knowledge and influences of teacher course and practice school. *J. Sci. Educ.* 11 76–79.

[B44] Nilsson P. Karlsson G. (2018). Capturing student teachers’ pedagogical content knowledge (PCK) using CoRes and digital technology. *Int. J. Sci. Educ.* 41 419–447. 10.1080/09500693.2018.1551642

[B45] Nilsson P. Loughran J. (2012). Exploring the development of pre-service science elementary teachers’ pedagogical content knowledge. *J. Sci. Teach. Educ.* 23 699–721. 10.1007/s10972011-9239-y

[B46] Padilla K. Ponce-de-Leon A. M. Rembado F. M. Garritz A. (2008). Undergraduate professors’ pedagogical content knowledge: the case of “amount of substance”. *Int. J. Sci. Educ.* 30 1389–1404. 10.1080/09500690802187033

[B47] Park S. Oliver J. S. (2008). Revisiting the conceptualization of pedagogical content knowledge (PCK): PCK as a conceptual tool to understand teachers as professionals. *Res. Sci. Educ.* 38 261–284. 10.1007/s11165-007-9049-6

[B48] Park S. Suh J. (2015). “From portraying toward assessing PCK: drivers, dilemmas, and directions for future research,” in *Re-Examining Pedagogical Content Knowledge in Science Education*, eds Berry A. Friedrichsen P. Loughran J. (Abingdon: Routledge), 104–119.

[B49] Peercy M. M. Sharkey J. (2020). Missing a S-STEP? How self-study of teacher education practice can support the language teacher education knowledge base. *Lang. Teach. Res.* 24 105–115. 10.1177/1362168818777526

[B50] Riedler M. Eryaman M. Y. (2016). Complexity, diversity and ambiguity in teaching and teacher education: practical wisdom, pedagogical fitness and tact of teaching. *Int. J. Prog. Educ.* 12 172–186.

[B51] Rollnick M. Bennett J. Rhemtula M. Dharsey N. Ndlovu T. (2008). The place of subject matter knowledge in pedagogical content knowledge: a case study of South African teachers teaching the amount of substance and chemical equilibrium. *Int. J. Sci. Educ.* 30 1365–1387. 10.1080/09500690802187025

[B52] Russell T. Martin A. K. (2014). “Learning to teach science,” in *Handbook of Research in Science Education*, eds Lederman N. G. Abell S. K. (Abingdon: Routledge), 871–888.

[B53] Schneider R. M. Plasman K. (2011). Science teacher learning progressions. *Rev. Educ. Res.* 81 530–565.

[B54] Shulman L. S. (1986). Those who understand: knowledge growth in teaching. *Educ. Res.* 15 4–14. 10.3102/0013189x015002004

[B55] Shulman L. S. (1987). Knowledge and teaching: foundations of new reform. *Harv. Educ. Rev.* 57 1–22. 10.4324/9781351233866-1

[B56] Sickel A. J. Friedrichsen P. (2013). Examining the evolution of education literature with a focus on teachers: major findings, goals for teacher preparation, and directions for future research. *Evol. Educ. Outreach* 6:23 10.1186/1936-6434-6-23

[B57] Smith S. P. Banilower E. R. (2015). “Assessing PCK: a new application of the uncertainty principle,” in *Re-Examining Pedagogical Content Knowledge in Science Education*, eds Berry A. Friedrichsen P. Loughran J. (Abingdon Oxon: Routledge), 88–103.

[B58] Strauss A. Corbin J. (1998). *Basic of Qualitative Research: Grounded Theory Procedures and Techniques.* Thousand Oaks, CA: Sage.

[B59] Sword H. (2011). “Archiving for the future: a longitudinal approach to evaluating a postgraduate certificate program,” in *Evaluating the Effectiveness of Academic Development: Principles and Practice*, ed. Stefani L. (New York, NY: Routledge), 127–132.

[B60] Tarone E. Allwright D. (2005). “Language teacher-learning and student language-learning: shaping the knowledge base,” in *Second Language Teacher Education: International Perspectives*, ed. Tedick D. J. (Mahwah, NJ: Lawrence Erlbaum), 5–23.

[B61] Thomson M. M. DiFrancesca D. Carrier S. Lee C. (2017). Teaching efficacy: exploring relationships between mathematics and science self-efficacy beliefs, PCK and domain knowledge among pre-service teachers from the United States. *Teach. Dev.* 21 1–20. 10.1080/13664530.2016.1204355

[B62] Torbeyns J. Verbruggen S. Depaepe F. (2019). Pedagogical content knowledge in pre-service preschool teachers and its association with opportunities to learn during teacher training. *ZDM Math. Educ.* 52 269–280. 10.1007/s11858-019-01088-y

[B63] Valk T. V. D. Broekman H. (1999). The lesson preparation method: a way of investigation pre-service teachers’ pedagogical content knowledge. *Eur. J. Teach. Educ.* 22 11–22. 10.1080/0261976990220102

[B64] Veal W. R. Makinster J. (1999). Pedagogical content knowledge taxonomies. *Electron. J. Sci. Educ.* 3 1–18.

[B65] Yang H. (2021). Epistemic agency, a double-stimulation, and video-based learning: a formative intervention study in language teacher education. *System* 96:102401 10.1016/j.system.2020.102401

[B66] Yuan R. Zhang L. J. (2017). Exploring student teachers’ motivation change in initial teacher education: a Chinese perspective. *Teach. Teach. Educ.* 61 142–152. 10.1016/j.tate.2016.10.010

[B67] Zarrinabadi N. Tavakoli M. (2017). Exploring motivational surges among Iranian EFL teacher trainees: directed motivational currents in focus. *TESOL Q.* 51 155–166. 10.1007/978-3-030-28380-3_7

[B68] Zeichner K. Conklin H. (2005). “Teacher education programs,” in *Studying Teacher Education*, eds Cochran Smith M. Zeichner K. (New York, NY: Routledge), 645–735.

[B69] Zhang L. J. Ben Said S. (2014). “Toward a global understanding of local initiatives in language teaching and teacher education: global rules, local roles,” in *Language Teachers and Teaching: Global Perspectives, Local Initiatives*, eds Ben Said S. Zhang L. J. (New York, NY: Routledge), xviii–xxx.

